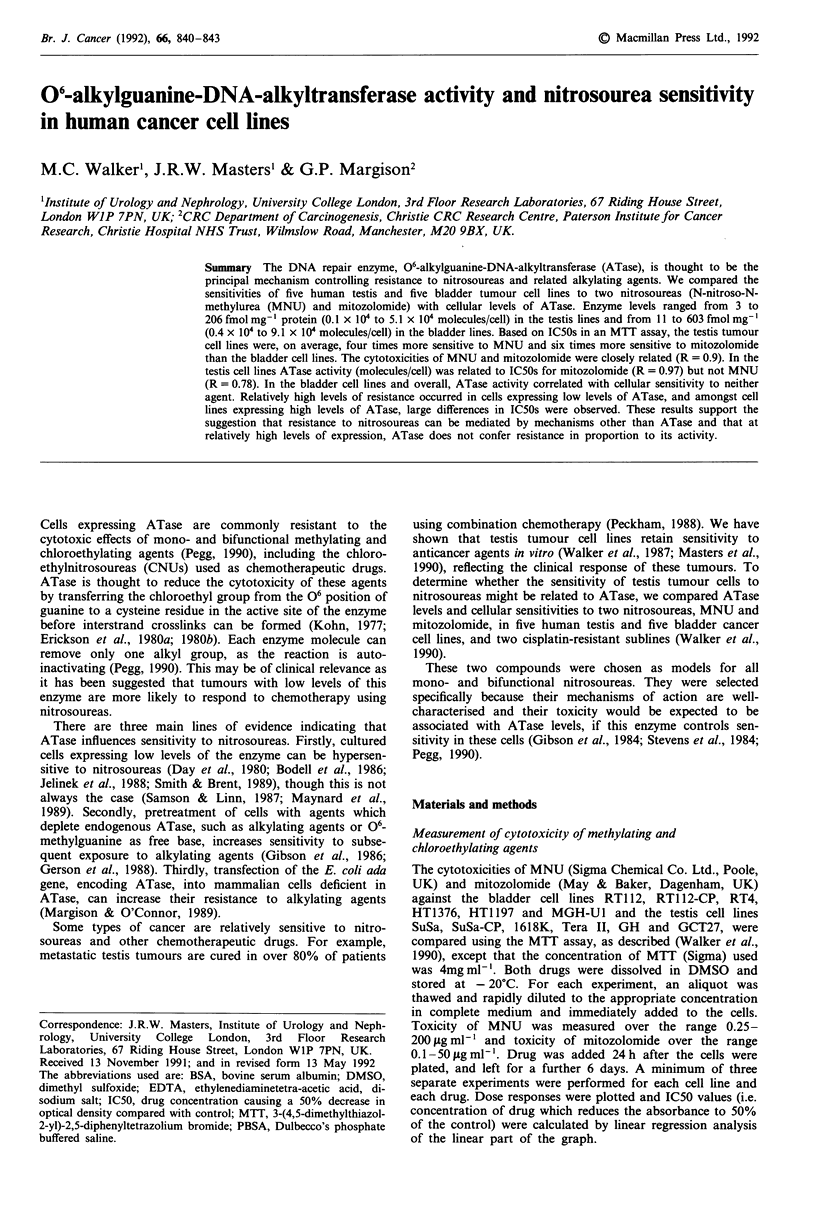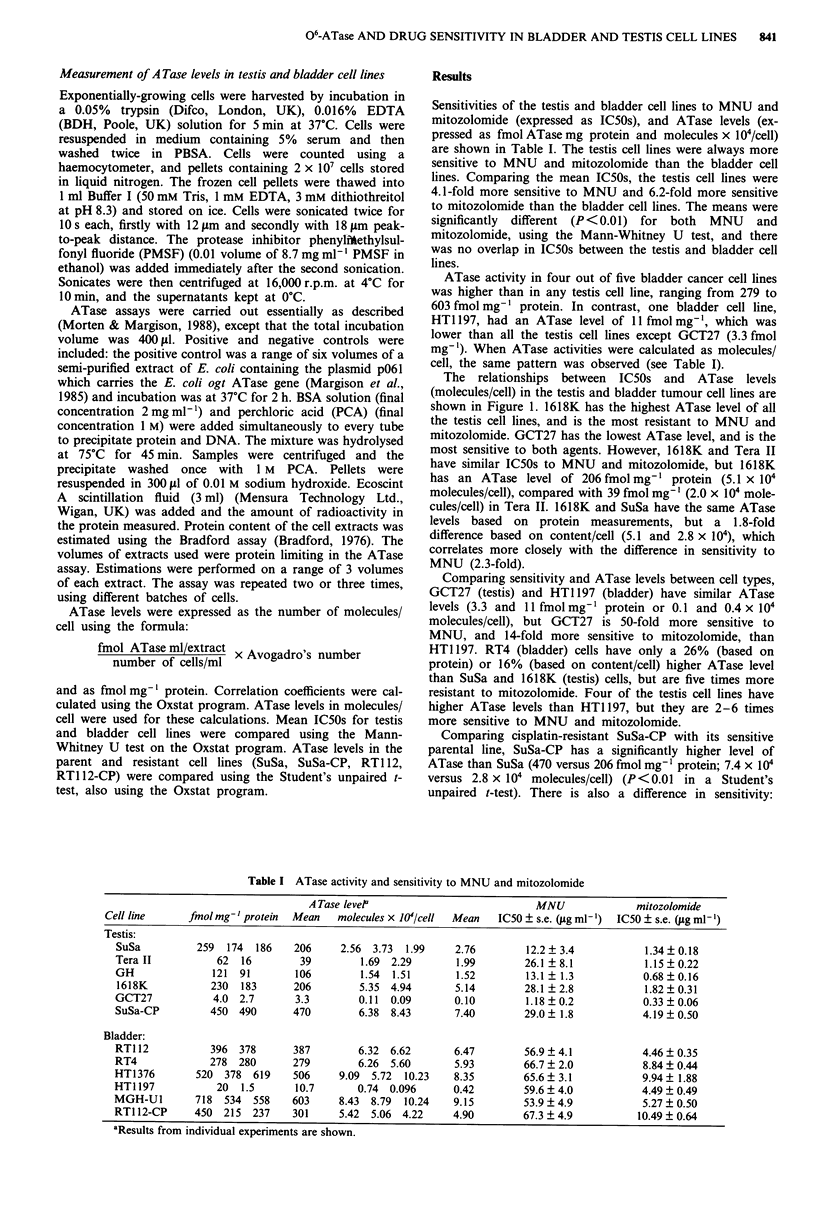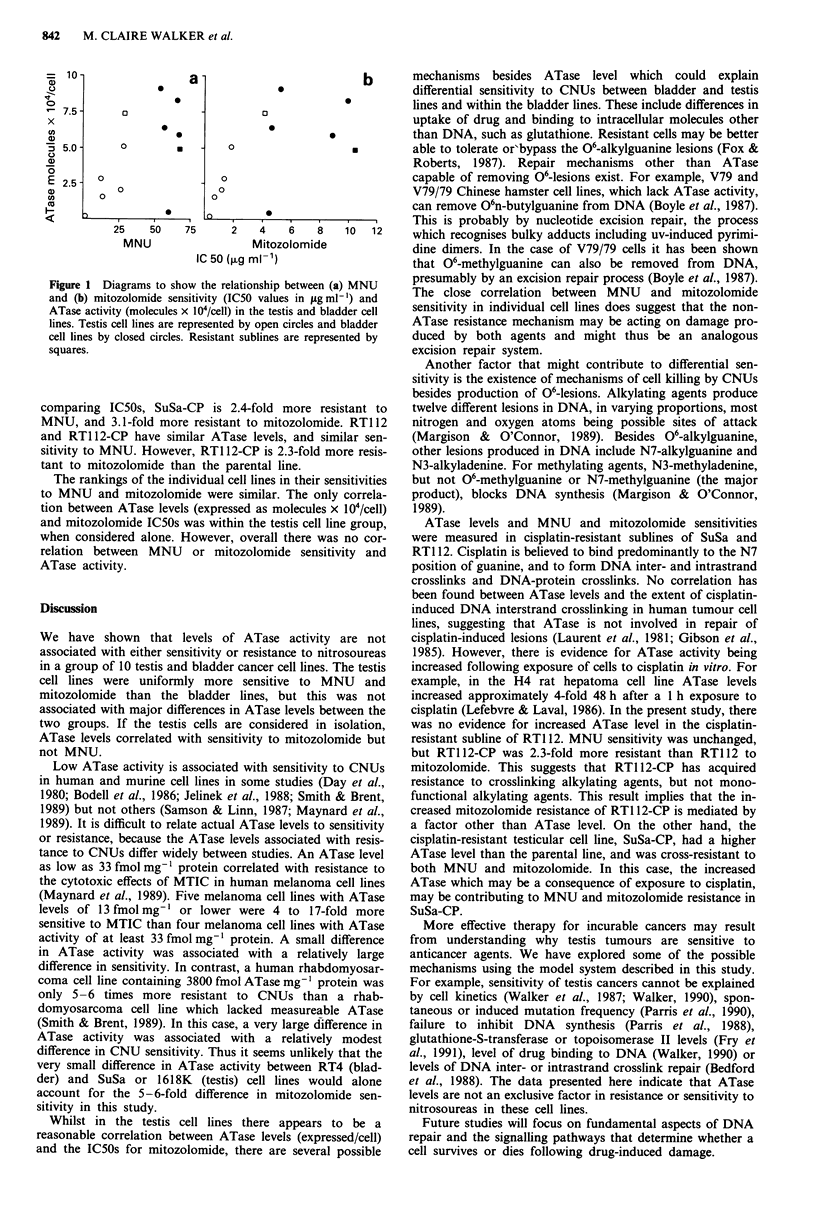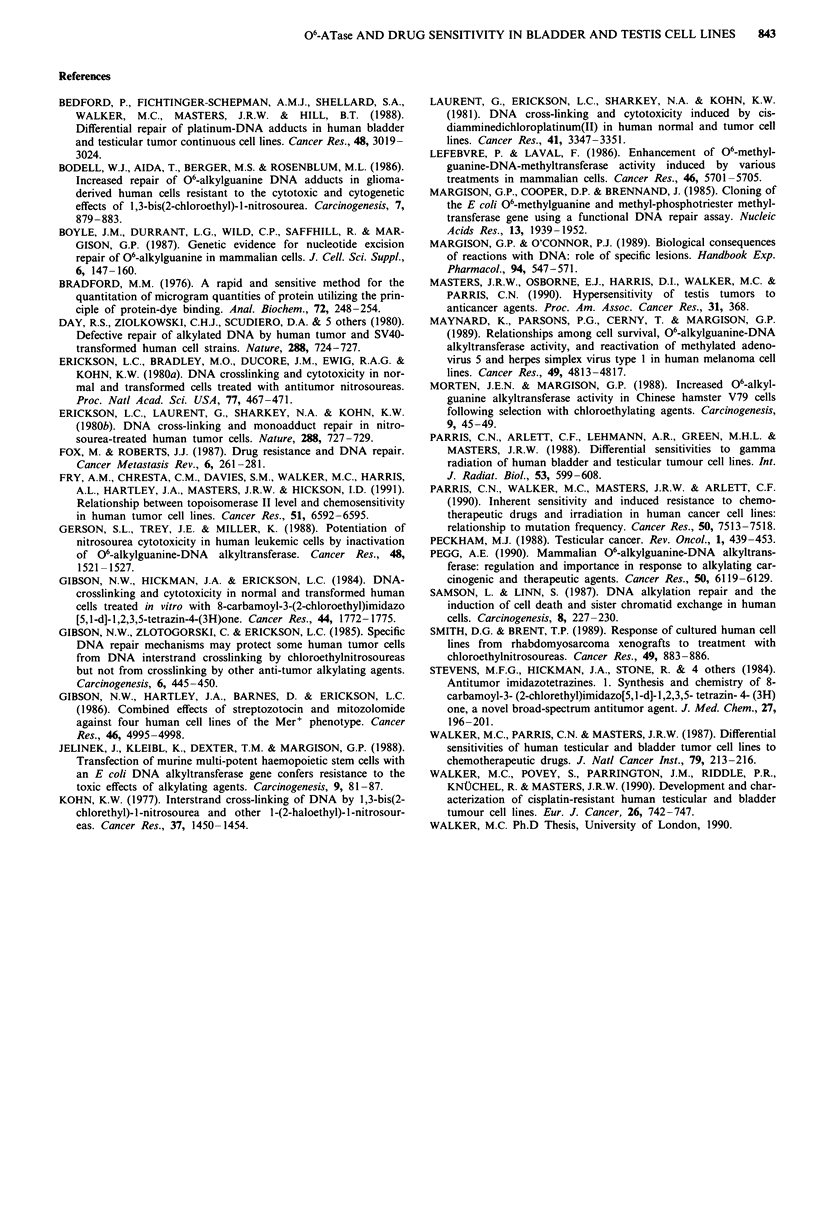# O6-alkylguanine-DNA-alkyltransferase activity and nitrosourea sensitivity in human cancer cell lines.

**DOI:** 10.1038/bjc.1992.370

**Published:** 1992-11

**Authors:** M. C. Walker, J. R. Masters, G. P. Margison

**Affiliations:** Institute of Urology and Nephrology, University College London, UK.

## Abstract

The DNA repair enzyme, O6-alkylguanine-DNA-alkyltransferase (ATase), is thought to be the principal mechanism controlling resistance to nitrosoureas and related alkylating agents. We compared the sensitivities of five human testis and five bladder tumour cell lines to two nitrosoureas (N-nitroso-N-methylurea (MNU) and mitozolomide) with cellular levels of ATase. Enzyme levels ranged from 3 to 206 fmol mg-1 protein (0.1 x 10(4) to 5.1 x 10(4) molecules/cell) in the testis lines and from 11 to 603 fmol mg-1 (0.4 x 10(4) to 9.1 x 10(4) molecules/cell) in the bladder lines. Based on IC50s in an MTT assay, the testis tumour cell lines were, on average, four times more sensitive to MNU and six times more sensitive to mitozolomide than the bladder cell lines. The cytotoxicities of MNU and mitozolomide were closely related (R = 0.9). In the testis cell lines ATase activity (molecules/cell) was related to IC50s for mitozolomide (R = 0.97) but not MNU (R = 0.78). In the bladder cell lines and overall, ATase activity correlated with cellular sensitivity to neither agent. Relatively high levels of resistance occurred in cells expressing low levels of ATase, and amongst cell lines expressing high levels of ATase, large differences in IC50s were observed. These results support the suggestion that resistance to nitrosoureas can be mediated by mechanisms other than ATase and that at relatively high levels of expression, ATase does not confer resistance in proportion to its activity.


Br. J. Cancer (1992), 66, 840-843 © Macmillan Press Ltd., 1992

### $O^6$-alkylguanine-DNA-alkyltransferase activity and nitrosourea sensitivity in human cancer cell lines

M.C. Walker[1], J.R.W. Masters[1] & G.P. Margison[2]

[1]Institute of Urology and Nephrology, University College London, 3rd Floor Research Laboratories, 67 Riding House Street, London W1P 7PN, UK; [2]CRC Department of Carcinogenesis, Christie CRC Research Centre, Paterson Institute for Cancer Research, Christie Hospital NHS Trust, Wilmslow Road, Manchester, M20 9BX, UK.

**Summary** The DNA repair enzyme, $O^6$-alkylguanine-DNA-alkyltransferase (ATase), is thought to be the principal mechanism controlling resistance to nitrosoureas and related alkylating agents. We compared the sensitivities of five human testis and five bladder tumour cell lines to two nitrosoureas (N-nitroso-N-methylurea (MNU) and mitozolomide) with cellular levels of ATase. Enzyme levels ranged from 3 to 206 fmol mg$^{-1}$ protein ($0.1 \times 10^4$ to $5.1 \times 10^4$ molecules/cell) in the testis lines and from 11 to 603 fmol mg$^{-1}$ ($0.4 \times 10^4$ to $9.1 \times 10^4$ molecules/cell) in the bladder lines. Based on IC50s in an MTT assay, the testis tumour cell lines were, on average, four times more sensitive to MNU and six times more sensitive to mitozolomide than the bladder cell lines. The cytotoxicities of MNU and mitozolomide were closely related ($R = 0.9$). In the testis cell lines ATase activity (molecules/cell) was related to IC50s for mitozolomide ($R = 0.97$) but not MNU ($R = 0.78$). In the bladder cell lines and overall, ATase activity correlated with cellular sensitivity to neither agent. Relatively high levels of resistance occurred in cells expressing low levels of ATase, and amongst cell lines expressing high levels of ATase, large differences in IC50s were observed. These results support the suggestion that resistance to nitrosoureas can be mediated by mechanisms other than ATase and that at relatively high levels of expression, ATase does not confer resistance in proportion to its activity.

Cells expressing ATase are commonly resistant to the cytotoxic effects of mono- and bifunctional methylating and chloroethylating agents (Pegg, 1990), including the chloroethylnitrosoureas (CNUs) used as chemotherapeutic drugs. ATase is thought to reduce the cytotoxicity of these agents by transferring the chloroethyl group from the $O^6$ position of guanine to a cysteine residue in the active site of the enzyme before interstrand crosslinks can be formed (Kohn, 1977; Erickson *et al.*, 1980*a*; 1980*b*). Each enzyme molecule can remove only one alkyl group, as the reaction is auto-inactivating (Pegg, 1990). This may be of clinical relevance as it has been suggested that tumours with low levels of this enzyme are more likely to respond to chemotherapy using nitrosoureas.

There are three main lines of evidence indicating that ATase influences sensitivity to nitrosoureas. Firstly, cultured cells expressing low levels of the enzyme can be hypersensitive to nitrosoureas (Day *et al.*, 1980; Bodell *et al.*, 1986; Jelinek *et al.*, 1988; Smith & Brent, 1989), though this is not always the case (Samson & Linn, 1987; Maynard *et al.*, 1989). Secondly, pretreatment of cells with agents which deplete endogenous ATase, such as alkylating agents or $O^6$-methylguanine as free base, increases sensitivity to subsequent exposure to alkylating agents (Gibson *et al.*, 1986; Gerson *et al.*, 1988). Thirdly, transfection of the *E. coli ada* gene, encoding ATase, into mammalian cells deficient in ATase, can increase their resistance to alkylating agents (Margison & O'Connor, 1989).

Some types of cancer are relatively sensitive to nitrosoureas and other chemotherapeutic drugs. For example, metastatic testis tumours are cured in over 80% of patients using combination chemotherapy (Peckham, 1988). We have shown that testis tumour cell lines retain sensitivity to anticancer agents *in vitro* (Walker *et al.*, 1987; Masters *et al.*, 1990), reflecting the clinical response of these tumours. To determine whether the sensitivity of testis tumour cells to nitrosoureas might be related to ATase, we compared ATase levels and cellular sensitivities to two nitrosoureas, MNU and mitozolomide, in five human testis and five bladder cancer cell lines, and two cisplatin-resistant sublines (Walker *et al.*, 1990).

These two compounds were chosen as models for all mono- and bifunctional nitrosoureas. They were selected specifically because their mechanisms of action are well-characterised and their toxicity would be expected to be associated with ATase levels, if this enzyme controls sensitivity in these cells (Gibson *et al.*, 1984; Stevens *et al.*, 1984; Pegg, 1990).

### Materials and methods

### *Measurement of cytotoxicity of methylating and chloroethylating agents*

The cytotoxicities of MNU (Sigma Chemical Co. Ltd., Poole, UK) and mitozolomide (May & Baker, Dagenham, UK) against the bladder cell lines RT112, RT112-CP, RT4, HT1376, HT1197 and MGH-U1 and the testis cell lines SuSa, SuSa-CP, 1618K, Tera II, GH and GCT27, were compared using the MTT assay, as described (Walker *et al.*, 1990), except that the concentration of MTT (Sigma) used was 4mg ml$^{-1}$. Both drugs were dissolved in DMSO and stored at $-20$°C. For each experiment, an aliquot was thawed and rapidly diluted to the appropriate concentration in complete medium and immediately added to the cells. Toxicity of MNU was measured over the range 0.25–200 $\mu$g ml$^{-1}$ and toxicity of mitozolomide over the range 0.1–50 $\mu$g ml$^{-1}$. Drug was added 24 h after the cells were plated, and left for a further 6 days. A minimum of three separate experiments were performed for each cell line and each drug. Dose responses were plotted and IC50 values (i.e. concentration of drug which reduces the absorbance to 50% of the control) were calculated by linear regression analysis of the linear part of the graph.

Correspondence: J.R.W. Masters, Institute of Urology and Nephrology, University College London, 3rd Floor Research Laboratories, 67 Riding House Street, London W1P 7PN, UK.
Received 13 November 1991; and in revised form 13 May 1992
The abbreviations used are: BSA, bovine serum albumin; DMSO, dimethyl sulfoxide; EDTA, ethylenediaminetetra-acetic acid, disodium salt; IC50, drug concentration causing a 50% decrease in optical density compared with control; MTT, 3-(4,5-dimethylthiazol-2-yl)-2,5-diphenyltetrazolium bromide; PBSA, Dulbecco's phosphate buffered saline.

*Measurement of ATase levels in testis and bladder cell lines*

Exponentially-growing cells were harvested by incubation in a 0.05% trypsin (Difco, London, UK), 0.016% EDTA (BDH, Poole, UK) solution for 5 min at 37°C. Cells were resuspended in medium containing 5% serum and then washed twice in PBSA. Cells were counted using a haemocytometer, and pellets containing $2 \times 10^7$ cells stored in liquid nitrogen. The frozen cell pellets were thawed into 1 ml Buffer I (50 mM Tris, 1 mM EDTA, 3 mM dithiothreitol at pH 8.3) and stored on ice. Cells were sonicated twice for 10 s each, firstly with 12 μm and secondly with 18 μm peak-to-peak distance. The protease inhibitor phenylmethylsulfonyl fluoride (PMSF) (0.01 volume of 8.7 mg ml$^{-1}$ PMSF in ethanol) was added immediately after the second sonication. Sonicates were then centrifuged at 16,000 r.p.m. at 4°C for 10 min, and the supernatants kept at 0°C.

ATase assays were carried out essentially as described (Morten & Margison, 1988), except that the total incubation volume was 400 μl. Positive and negative controls were included: the positive control was a range of six volumes of a semi-purified extract of *E. coli* containing the plasmid p061 which carries the *E. coli ogt* ATase gene (Margison *et al.*, 1985) and incubation was at 37°C for 2 h. BSA solution (final concentration 2 mg ml$^{-1}$) and perchloric acid (PCA) (final concentration 1 M) were added simultaneously to every tube to precipitate protein and DNA. The mixture was hydrolysed at 75°C for 45 min. Samples were centrifuged and the precipitate washed once with 1 M PCA. Pellets were resuspended in 300 μl of 0.01 M sodium hydroxide. Ecoscint A scintillation fluid (3 ml) (Mensura Technology Ltd., Wigan, UK) was added and the amount of radioactivity in the protein measured. Protein content of the cell extracts was estimated using the Bradford assay (Bradford, 1976). The volumes of extracts used were protein limiting in the ATase assay. Estimations were performed on a range of 3 volumes of each extract. The assay was repeated two or three times, using different batches of cells.

ATase levels were expressed as the number of molecules/cell using the formula:

$$\frac{\text{fmol ATase ml/extract}}{\text{number of cells/ml}} \times \text{Avogadro's number}$$

and as fmol mg$^{-1}$ protein. Correlation coefficients were calculated using the Oxstat program. ATase levels in molecules/cell were used for these calculations. Mean IC50s for testis and bladder cell lines were compared using the Mann-Whitney U test on the Oxstat program. ATase levels in the parent and resistant cell lines (SuSa, SuSa-CP, RT112, RT112-CP) were compared using the Student's unpaired *t*-test, also using the Oxstat program.

### Results

Sensitivities of the testis and bladder cell lines to MNU and mitozolomide (expressed as IC50s), and ATase levels (expressed as fmol ATase mg protein and molecules $\times 10^4$/cell) are shown in Table I. The testis cell lines were always more sensitive to MNU and mitozolomide than the bladder cell lines. Comparing the mean IC50s, the testis cell lines were 4.1-fold more sensitive to MNU and 6.2-fold more sensitive to mitozolomide than the bladder cell lines. The means were significantly different ($P < 0.01$) for both MNU and mitozolomide, using the Mann-Whitney U test, and there was no overlap in IC50s between the testis and bladder cell lines.

ATase activity in four out of five bladder cancer cell lines was higher than in any testis cell line, ranging from 279 to 603 fmol mg$^{-1}$ protein. In contrast, one bladder cell line, HT1197, had an ATase level of 11 fmol mg$^{-1}$, which was lower than all the testis cell lines except GCT27 (3.3 fmol mg$^{-1}$). When ATase activities were calculated as molecules/cell, the same pattern was observed (see Table I).

The relationships between IC50s and ATase levels (molecules/cell) in the testis and bladder tumour cell lines are shown in Figure 1. 1618K has the highest ATase level of all the testis cell lines, and is the most resistant to MNU and mitozolomide. GCT27 has the lowest ATase level, and is the most sensitive to both agents. However, 1618K and Tera II have similar IC50s to MNU and mitozolomide, but 1618K has an ATase level of 206 fmol mg$^{-1}$ protein ($5.1 \times 10^4$ molecules/cell), compared with 39 fmol mg$^{-1}$ ($2.0 \times 10^4$ molecules/cell) in Tera II. 1618K and SuSa have the same ATase levels based on protein measurements, but a 1.8-fold difference based on content/cell (5.1 and $2.8 \times 10^4$), which correlates more closely with the difference in sensitivity to MNU (2.3-fold).

Comparing sensitivity and ATase levels between cell types, GCT27 (testis) and HT1197 (bladder) have similar ATase levels (3.3 and 11 fmol mg$^{-1}$ protein or 0.1 and $0.4 \times 10^4$ molecules/cell), but GCT27 is 50-fold more sensitive to MNU, and 14-fold more sensitive to mitozolomide, than HT1197. RT4 (bladder) cells have only a 26% (based on protein) or 16% (based on content/cell) higher ATase level than SuSa and 1618K (testis) cells, but are five times more resistant to mitozolomide. Four of the testis cell lines have higher ATase levels than HT1197, but they are 2–6 times more sensitive to MNU and mitozolomide.

Comparing cisplatin-resistant SuSa-CP with its sensitive parental line, SuSa-CP has a significantly higher level of ATase than SuSa (470 versus 206 fmol mg$^{-1}$ protein; $7.4 \times 10^4$ versus $2.8 \times 10^4$ molecules/cell) ($P < 0.01$ in a Student's unpaired *t*-test). There is also a difference in sensitivity:

**Table I** ATase activity and sensitivity to MNU and mitozolomide

| Cell line | *ATase level*[a] *fmol mg$^{-1}$ protein* | *Mean* | *molecules × $10^4$/cell* | *Mean* | *MNU* IC50 ± s.e. (μg ml$^{-1}$) | *mitozolomide* IC50 ± s.e. (μg ml$^{-1}$) |
|---|---|---|---|---|---|---|
| Testis: | | | | | | |
| SuSa | 259 174 186 | 206 | 2.56 3.73 1.99 | 2.76 | 12.2 ± 3.4 | 1.34 ± 0.18 |
| Tera II | 62 16 | 39 | 1.69 2.29 | 1.99 | 26.1 ± 8.1 | 1.15 ± 0.22 |
| GH | 121 91 | 106 | 1.54 1.51 | 1.52 | 13.1 ± 1.3 | 0.68 ± 0.16 |
| 1618K | 230 183 | 206 | 5.35 4.94 | 5.14 | 28.1 ± 2.8 | 1.82 ± 0.31 |
| GCT27 | 4.0 2.7 | 3.3 | 0.11 0.09 | 0.10 | 1.18 ± 0.2 | 0.33 ± 0.06 |
| SuSa-CP | 450 490 | 470 | 6.38 8.43 | 7.40 | 29.0 ± 1.8 | 4.19 ± 0.50 |
| Bladder: | | | | | | |
| RT112 | 396 378 | 387 | 6.32 6.62 | 6.47 | 56.9 ± 4.1 | 4.46 ± 0.35 |
| RT4 | 278 280 | 279 | 6.26 5.60 | 5.93 | 66.7 ± 2.0 | 8.84 ± 0.44 |
| HT1376 | 520 378 619 | 506 | 9.09 5.72 10.23 | 8.35 | 65.6 ± 3.1 | 9.94 ± 1.88 |
| HT1197 | 20 1.5 | 10.7 | 0.74 0.096 | 0.42 | 59.6 ± 4.0 | 4.49 ± 0.49 |
| MGH-U1 | 718 534 558 | 603 | 8.43 8.79 10.24 | 9.15 | 53.9 ± 4.9 | 5.27 ± 0.50 |
| RT112-CP | 450 215 237 | 301 | 5.42 5.06 4.22 | 4.90 | 67.3 ± 4.9 | 10.49 ± 0.64 |

[a]Results from individual experiments are shown.

**Figure 1** Diagrams to show the relationship between (a) MNU and (b) mitozolomide sensitivity (IC50 values in $\mu g\, ml^{-1}$) and ATase activity (molecules $\times 10^4$/cell) in the testis and bladder cell lines. Testis cell lines are represented by open circles and bladder cell lines by closed circles. Resistant sublines are represented by squares.

comparing IC50s, SuSa-CP is 2.4-fold more resistant to MNU, and 3.1-fold more resistant to mitozolomide. RT112 and RT112-CP have similar ATase levels, and similar sensitivity to MNU. However, RT112-CP is 2.3-fold more resistant to mitozolomide than the parental line.

The rankings of the individual cell lines in their sensitivities to MNU and mitozolomide were similar. The only correlation between ATase levels (expressed as molecules $\times 10^4$/cell) and mitozolomide IC50s was within the testis cell line group, when considered alone. However, overall there was no correlation between MNU or mitozolomide sensitivity and ATase activity.

### Discussion

We have shown that levels of ATase activity are not associated with either sensitivity or resistance to nitrosoureas in a group of 10 testis and bladder cancer cell lines. The testis cell lines were uniformly more sensitive to MNU and mitozolomide than the bladder lines, but this was not associated with major differences in ATase levels between the two groups. If the testis cells are considered in isolation, ATase levels correlated with sensitivity to mitozolomide but not MNU.

Low ATase activity is associated with sensitivity to CNUs in human and murine cell lines in some studies (Day *et al.*, 1980; Bodell *et al.*, 1986; Jelinek *et al.*, 1988; Smith & Brent, 1989) but not others (Samson & Linn, 1987; Maynard *et al.*, 1989). It is difficult to relate actual ATase levels to sensitivity or resistance, because the ATase levels associated with resistance to CNUs differ widely between studies. An ATase level as low as 33 $fmol\, mg^{-1}$ protein correlated with resistance to the cytotoxic effects of MTIC in human melanoma cell lines (Maynard *et al.*, 1989). Five melanoma cell lines with ATase levels of 13 $fmol\, mg^{-1}$ or lower were 4 to 17-fold more sensitive to MTIC than four melanoma cell lines with ATase activity of at least 33 $fmol\, mg^{-1}$ protein. A small difference in ATase activity was associated with a relatively large difference in sensitivity. In contrast, a human rhabdomyosarcoma cell line containing 3800 fmol ATase $mg^{-1}$ protein was only 5–6 times more resistant to CNUs than a rhabdomyosarcoma cell line which lacked measureable ATase (Smith & Brent, 1989). In this case, a very large difference in ATase activity was associated with a relatively modest difference in CNU sensitivity. Thus it seems unlikely that the very small difference in ATase activity between RT4 (bladder) and SuSa or 1618K (testis) cell lines would alone account for the 5–6-fold difference in mitozolomide sensitivity in this study.

Whilst in the testis cell lines there appears to be a reasonable correlation between ATase levels (expressed/cell) and the IC50s for mitozolomide, there are several possible mechanisms besides ATase level which could explain differential sensitivity to CNUs between bladder and testis lines and within the bladder lines. These include differences in uptake of drug and binding to intracellular molecules other than DNA, such as glutathione. Resistant cells may be better able to tolerate or bypass the $O^6$-alkylguanine lesions (Fox & Roberts, 1987). Repair mechanisms other than ATase capable of removing $O^6$-lesions exist. For example, V79 and V79/79 Chinese hamster cell lines, which lack ATase activity, can remove $O^6$n-butylguanine from DNA (Boyle *et al.*, 1987). This is probably by nucleotide excision repair, the process which recognises bulky adducts including uv-induced pyrimidine dimers. In the case of V79/79 cells it has been shown that $O^6$-methylguanine can also be removed from DNA, presumably by an excision repair process (Boyle *et al.*, 1987). The close correlation between MNU and mitozolomide sensitivity in individual cell lines does suggest that the non-ATase resistance mechanism may be acting on damage produced by both agents and might thus be an analogous excision repair system.

Another factor that might contribute to differential sensitivity is the existence of mechanisms of cell killing by CNUs besides production of $O^6$-lesions. Alkylating agents produce twelve different lesions in DNA, in varying proportions, most nitrogen and oxygen atoms being possible sites of attack (Margison & O'Connor, 1989). Besides $O^6$-alkylguanine, other lesions produced in DNA include N7-alkylguanine and N3-alkyladenine. For methylating agents, N3-methyladenine, but not $O^6$-methylguanine or N7-methylguanine (the major product), blocks DNA synthesis (Margison & O'Connor, 1989).

ATase levels and MNU and mitozolomide sensitivities were measured in cisplatin-resistant sublines of SuSa and RT112. Cisplatin is believed to bind predominantly to the N7 position of guanine, and to form DNA inter- and intrastrand crosslinks and DNA-protein crosslinks. No correlation has been found between ATase levels and the extent of cisplatin-induced DNA interstrand crosslinking in human tumour cell lines, suggesting that ATase is not involved in repair of cisplatin-induced lesions (Laurent *et al.*, 1981; Gibson *et al.*, 1985). However, there is evidence for ATase activity being increased following exposure of cells to cisplatin *in vitro*. For example, in the H4 rat hepatoma cell line ATase levels increased approximately 4-fold 48 h after a 1 h exposure to cisplatin (Lefebvre & Laval, 1986). In the present study, there was no evidence for increased ATase level in the cisplatin-resistant subline of RT112. MNU sensitivity was unchanged, but RT112-CP was 2.3-fold more resistant than RT112 to mitozolomide. This suggests that RT112-CP has acquired resistance to crosslinking alkylating agents, but not monofunctional alkylating agents. This result implies that the increased mitozolomide resistance of RT112-CP is mediated by a factor other than ATase level. On the other hand, the cisplatin-resistant testicular cell line, SuSa-CP, had a higher ATase level than the parental line, and was cross-resistant to both MNU and mitozolomide. In this case, the increased ATase which may be a consequence of exposure to cisplatin, may be contributing to MNU and mitozolomide resistance in SuSa-CP.

More effective therapy for incurable cancers may result from understanding why testis tumours are sensitive to anticancer agents. We have explored some of the possible mechanisms using the model system described in this study. For example, sensitivity of testis cancers cannot be explained by cell kinetics (Walker *et al.*, 1987; Walker, 1990), spontaneous or induced mutation frequency (Parris *et al.*, 1990), failure to inhibit DNA synthesis (Parris *et al.*, 1988), glutathione-S-transferase or topoisomerase II levels (Fry *et al.*, 1991), level of drug binding to DNA (Walker, 1990) or levels of DNA inter- or intrastrand crosslink repair (Bedford *et al.*, 1988). The data presented here indicate that ATase levels are not an exclusive factor in resistance or sensitivity to nitrosoureas in these cell lines.

Future studies will focus on fundamental aspects of DNA repair and the signalling pathways that determine whether a cell survives or dies following drug-induced damage.

### References

BEDFORD, P., FICHTINGER-SCHEPMAN, A.M.J., SHELLARD, S.A., WALKER, M.C., MASTERS, J.R.W. & HILL, B.T. (1988). Differential repair of platinum-DNA adducts in human bladder and testicular tumor continuous cell lines. *Cancer Res.*, **48**, 3019–3024.

BODELL, W.J., AIDA, T., BERGER, M.S. & ROSENBLUM, M.L. (1986). Increased repair of $O^6$-alkylguanine DNA adducts in glioma-derived human cells resistant to the cytotoxic and cytogenetic effects of 1,3-bis(2-chloroethyl)-1-nitrosourea. *Carcinogenesis*, **7**, 879–883.

BOYLE, J.M., DURRANT, L.G., WILD, C.P., SAFFHILL, R. & MARGISON, G.P. (1987). Genetic evidence for nucleotide excision repair of $O^6$-alkylguanine in mammalian cells. *J. Cell. Sci. Suppl.*, **6**, 147–160.

BRADFORD, M.M. (1976). A rapid and sensitive method for the quantitation of microgram quantities of protein utilizing the principle of protein-dye binding. *Anal. Biochem.*, **72**, 248–254.

DAY, R.S., ZIOLKOWSKI, C.H.J., SCUDIERO, D.A. & 5 others (1980). Defective repair of alkylated DNA by human tumor and SV40-transformed human cell strains. *Nature*, **288**, 724–727.

ERICKSON, L.C., BRADLEY, M.O., DUCORE, J.M., EWIG, R.A.G. & KOHN, K.W. (1980*a*). DNA crosslinking and cytotoxicity in normal and transformed cells treated with antitumor nitrosoureas. *Proc. Natl Acad. Sci. USA*, **77**, 467–471.

ERICKSON, L.C., LAURENT, G., SHARKEY, N.A. & KOHN, K.W. (1980*b*). DNA cross-linking and monoadduct repair in nitrosourea-treated human tumor cells. *Nature*, **288**, 727–729.

FOX, M. & ROBERTS, J.J. (1987). Drug resistance and DNA repair. *Cancer Metastasis Rev.*, **6**, 261–281.

FRY, A.M., CHRESTA, C.M., DAVIES, S.M., WALKER, M.C., HARRIS, A.L., HARTLEY, J.A., MASTERS, J.R.W. & HICKSON, I.D. (1991). Relationship between topoisomerase II level and chemosensitivity in human tumor cell lines. *Cancer Res.*, **51**, 6592–6595.

GERSON, S.L., TREY, J.E. & MILLER, K. (1988). Potentiation of nitrosourea cytotoxicity in human leukemic cells by inactivation of $O^6$-alkylguanine-DNA alkyltransferase. *Cancer Res.*, **48**, 1521–1527.

GIBSON, N.W., HICKMAN, J.A. & ERICKSON, L.C. (1984). DNA-crosslinking and cytotoxicity in normal and transformed human cells treated *in vitro* with 8-carbamoyl-3-(2-chloroethyl)imidazo [5,1-d]-1,2,3,5-tetrazin-4-(3H)one. *Cancer Res.*, **44**, 1772–1775.

GIBSON, N.W., ZLOTOGORSKI, C. & ERICKSON, L.C. (1985). Specific DNA repair mechanisms may protect some human tumor cells from DNA interstrand crosslinking by chloroethylnitrosoureas but not from crosslinking by other anti-tumor alkylating agents. *Carcinogenesis*, **6**, 445–450.

GIBSON, N.W., HARTLEY, J.A., BARNES, D. & ERICKSON, L.C. (1986). Combined effects of streptozotocin and mitozolomide against four human cell lines of the $Mer^+$ phenotype. *Cancer Res.*, **46**, 4995–4998.

JELINEK, J., KLEIBL, K., DEXTER, T.M. & MARGISON, G.P. (1988). Transfection of murine multi-potent haemopoietic stem cells with an *E coli* DNA alkyltransferase gene confers resistance to the toxic effects of alkylating agents. *Carcinogenesis*, **9**, 81–87.

KOHN, K.W. (1977). Interstrand cross-linking of DNA by 1,3-bis(2-chlorethyl)-1-nitrosourea and other 1-(2-haloethyl)-1-nitrosoureas. *Cancer Res.*, **37**, 1450–1454.

LAURENT, G., ERICKSON, L.C., SHARKEY, N.A. & KOHN, K.W. (1981). DNA cross-linking and cytotoxicity induced by cis-diamminedichloroplatinum(II) in human normal and tumor cell lines. *Cancer Res.*, **41**, 3347–3351.

LEFEBVRE, P. & LAVAL, F. (1986). Enhancement of $O^6$-methylguanine-DNA-methyltransferase activity induced by various treatments in mammalian cells. *Cancer Res.*, **46**, 5701–5705.

MARGISON, G.P., COOPER, D.P. & BRENNAND, J. (1985). Cloning of the *E coli* $O^6$-methylguanine and methyl-phosphotriester methyltransferase gene using a functional DNA repair assay. *Nucleic Acids Res.*, **13**, 1939–1952.

MARGISON, G.P. & O'CONNOR, P.J. (1989). Biological consequences of reactions with DNA: role of specific lesions. *Handbook Exp. Pharmacol.*, **94**, 547–571.

MASTERS, J.R.W., OSBORNE, E.J., HARRIS, D.I., WALKER, M.C. & PARRIS, C.N. (1990). Hypersensitivity of testis tumors to anticancer agents. *Proc. Am. Assoc. Cancer Res.*, **31**, 368.

MAYNARD, K., PARSONS, P.G., CERNY, T. & MARGISON, G.P. (1989). Relationships among cell survival, $O^6$-alkylguanine-DNA alkyltransferase activity, and reactivation of methylated adenovirus 5 and herpes simplex virus type 1 in human melanoma cell lines. *Cancer Res.*, **49**, 4813–4817.

MORTEN, J.E.N. & MARGISON, G.P. (1988). Increased $O^6$-alkylguanine alkyltransferase activity in Chinese hamster V79 cells following selection with chloroethylating agents. *Carcinogenesis*, **9**, 45–49.

PARRIS, C.N., ARLETT, C.F., LEHMANN, A.R., GREEN, M.H.L. & MASTERS, J.R.W. (1988). Differential sensitivities to gamma radiation of human bladder and testicular tumour cell lines. *Int. J. Radiat. Biol.*, **53**, 599–608.

PARRIS, C.N., WALKER, M.C., MASTERS, J.R.W. & ARLETT, C.F. (1990). Inherent sensitivity and induced resistance to chemotherapeutic drugs and irradiation in human cancer cell lines: relationship to mutation frequency. *Cancer Res.*, **50**, 7513–7518.

PECKHAM, M.J. (1988). Testicular cancer. *Rev. Oncol.*, **1**, 439–453.

PEGG, A.E. (1990). Mammalian $O^6$-alkylguanine-DNA alkyltransferase: regulation and importance in response to alkylating carcinogenic and therapeutic agents. *Cancer Res.*, **50**, 6119–6129.

SAMSON, L. & LINN, S. (1987). DNA alkylation repair and the induction of cell death and sister chromatid exchange in human cells. *Carcinogenesis*, **8**, 227–230.

SMITH, D.G. & BRENT, T.P. (1989). Response of cultured human cell lines from rhabdomyosarcoma xenografts to treatment with chloroethylnitrosoureas. *Cancer Res.*, **49**, 883–886.

STEVENS, M.F.G., HICKMAN, J.A., STONE, R. & 4 others (1984). Antitumor imidazotetrazines. 1. Synthesis and chemistry of 8-carbamoyl-3- (2-chlorethyl)imidazo[5,1-d]-1,2,3,5- tetrazin- 4- (3H) one, a novel broad-spectrum antitumor agent. *J. Med. Chem.*, **27**, 196–201.

WALKER, M.C., PARRIS, C.N. & MASTERS, J.R.W. (1987). Differential sensitivities of human testicular and bladder tumor cell lines to chemotherapeutic drugs. *J. Natl Cancer Inst.*, **79**, 213–216.

WALKER, M.C., POVEY, S., PARRINGTON, J.M., RIDDLE, P.R., KNÜCHEL, R. & MASTERS, J.R.W. (1990). Development and characterization of cisplatin-resistant human testicular and bladder tumour cell lines. *Eur. J. Cancer*, **26**, 742–747.

WALKER, M.C. Ph.D Thesis, University of London, 1990.